# Rotation axis demultiplexer enabling simultaneous computed tomography of multiple samples

**DOI:** 10.1016/j.mex.2016.04.005

**Published:** 2016-04-18

**Authors:** Pavel Trtik, Fabian Geiger, Jan Hovind, Udo Lang, Eberhard Lehmann, Peter Vontobel, Steven Peetermans

**Affiliations:** a Neutron Imaging and Activation Group, Laboratory for Neutron Scattering and Imaging, Paul Scherrer Institut, 5232 Villigen PSI, Switzerland; b Lucerne School of Engineering and Architecture, CC Mechanical Systems, Technikumstrasse 21, 6048 Horw, Switzerland

**Keywords:** Computed tomography, multiaxial simultaneous tomography, neutron imaging, computed tomgraphy

## Abstract

This paper describes a device that allows for simultaneous tomographic imaging of samples on three independent rotational axes. This rotation axis demultiplexer (POLYTOM) is equipped with anti-backlash gears and placed on a standard sample rotation stage thus allowing for the transformation of the input rotation axis onto two additional parallel vertical axes. Consequently, three times the number of samples can be investigated within a given time period, thereby reducing the acquisition time of multiple sample tomographic investigations by a factor of three. The results of our pilot experiments using neutron tomographic imaging are presented. We foresee that the device will be of particular use for tomographic imaging of elongated samples at low-flux (e.g. neutron) sources; however, its use for the more widespread types of imaging techniques (e.g. X-rays) is not ruled out. The highlights of this new device for the purpose of the (neutron) computed tomography are:

• Anti-backlash transformation of the input rotation onto two additional rotational axes.
• Reduction of the acquisition time of the multiple sample tomographic investigations by a factor of three.
• Low-cost.

## Graphical abstract

**Figure:**
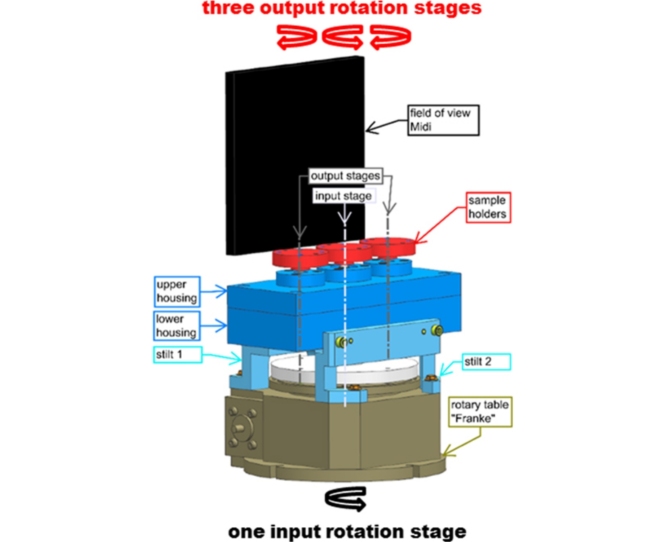

## Method details

The temporal resolution of some imaging facilities (e.g. those for neutron imaging) is limited by the relatively low flux of its sources. Even with the most powerful neutron sources it is common that the acquisition time for neutron radiographies is on the order of many seconds to minutes, while the time required for acquisition of entire neutron tomographies is on the order of tens of hours (e.g. [1]). The neutron flux consideration is even less favorable in the case of energy-selective neutron imaging [2] in which only fraction of the incoming polychromatic beam is utilized and thus the times needed for the statistically-relevant investigations might be even more demanding. Such time budgets therefore pose serious obstacles for investigations that require high throughput of samples (e.g. biological studies [3]).

While the size and shape of the field of view depend on a particular test arrangement, the typical field of view in (neutron) full-field imaging is given by the shape of the utilized detector. The routinely used charge-coupled devices (CCDs) or complementary metal-oxide-semiconductor (CMOS) cameras are usually in the shape of a square or low-aspect-ratio rectangle. Likewise, the available neutron beam profile at the detector position usually has similar vertical and horizontal dimensions.

For tomographic imaging, samples are placed on a rotational stage and radiographs are acquired from various angular positions. In the case that the samples are significantly smaller than the field of view, it is possible, and indeed a common practice, to stack the samples above each other and perform the tomography investigation of multiple samples in the same tomographic run. However, even when stacked in the vertical direction, the samples often fill up only a limited part of the available field of view in the horizontal direction [4]. This also holds true for samples with elongated shapes (e.g., swords [5], [6], cladding tubes [7], etc.), which intrinsically occupy only a limited part of the view in the horizontal direction. For such sample shapes and sizes, it would be rather advantageous to be able to perform simultaneous tomographic imaging of multiple samples by utilizing more of the detector area and, thus, the (neutron) flux more efficiently.

Tomographic sample stages are routinely equipped with a single rotational stage with its (usually vertical) axis of rotation parallel to the imaging plane (e.g., the scintillator screen). In this paper, we demonstrate that a rotation axis demultiplexer (POLYTOM) can be reliably used to transform the rotation from this single rotational axis onto two adjacent parallel axes. In this way, the field of view is divided into three independent vertical segments in which three tomographies can be run simultaneously. We tested the device using both neutron and X-ray imaging at the NEUTRA beamline, and we demonstrate that the three resulting tomographic datasets show no sign of any instability or irregularity artifacts due to its use.

## Methods

**Fig. 1:**
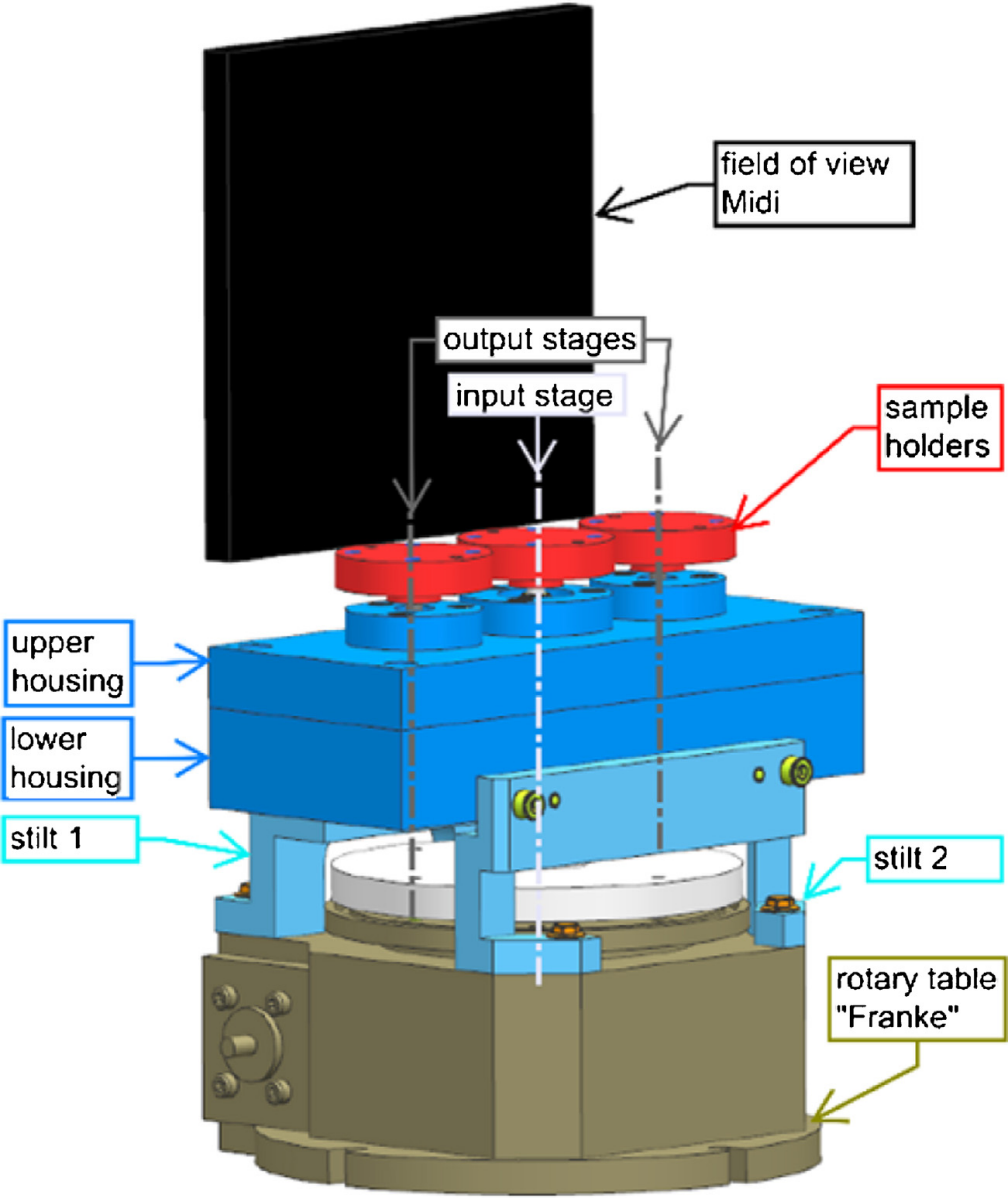
Rotation axis demultiplexer (POLYTOM): the new device (blue parts) is placed on an existing rotating stage (grey). The height of the sample holders (red) is determined such that the complete field of view can be used for experiments.

The POLYTOM device was designed with three actuated axes. In order to provide the highest possible precision, and hence obtain the highest resolution during tomography, two constraints were applied to the mechanical design: (i) minimization of tolerances, and (ii) no allowable backlash from the gears. It is worth pointing out here that the anti-backlash gears are particularly important for the tomographic experiments, during which the direction of the motion of the rotation stages changes (i.e. time series of tomographic experiments and/or any tomographic experiment using non-sequential data acquisition schemes [8]). We used the standard rotary table (type Franke LTB125, Franke GmbH, Aalen, Germany) employed at both the NEUTRA and ICON beamlines [9], [10] for neutron imaging at a spatial resolution of about 100 μm. We chose to space the three parallel axes 50 mm apart in order to distribute the axes uniformly over the most commonly utilized field of view (150 mm × 150 mm) at the NEUTRA beamline. Fig. 1 shows the final integration of the demultiplexer on the existing rotary Table in front of the beamline detector.

**Fig. 2:**
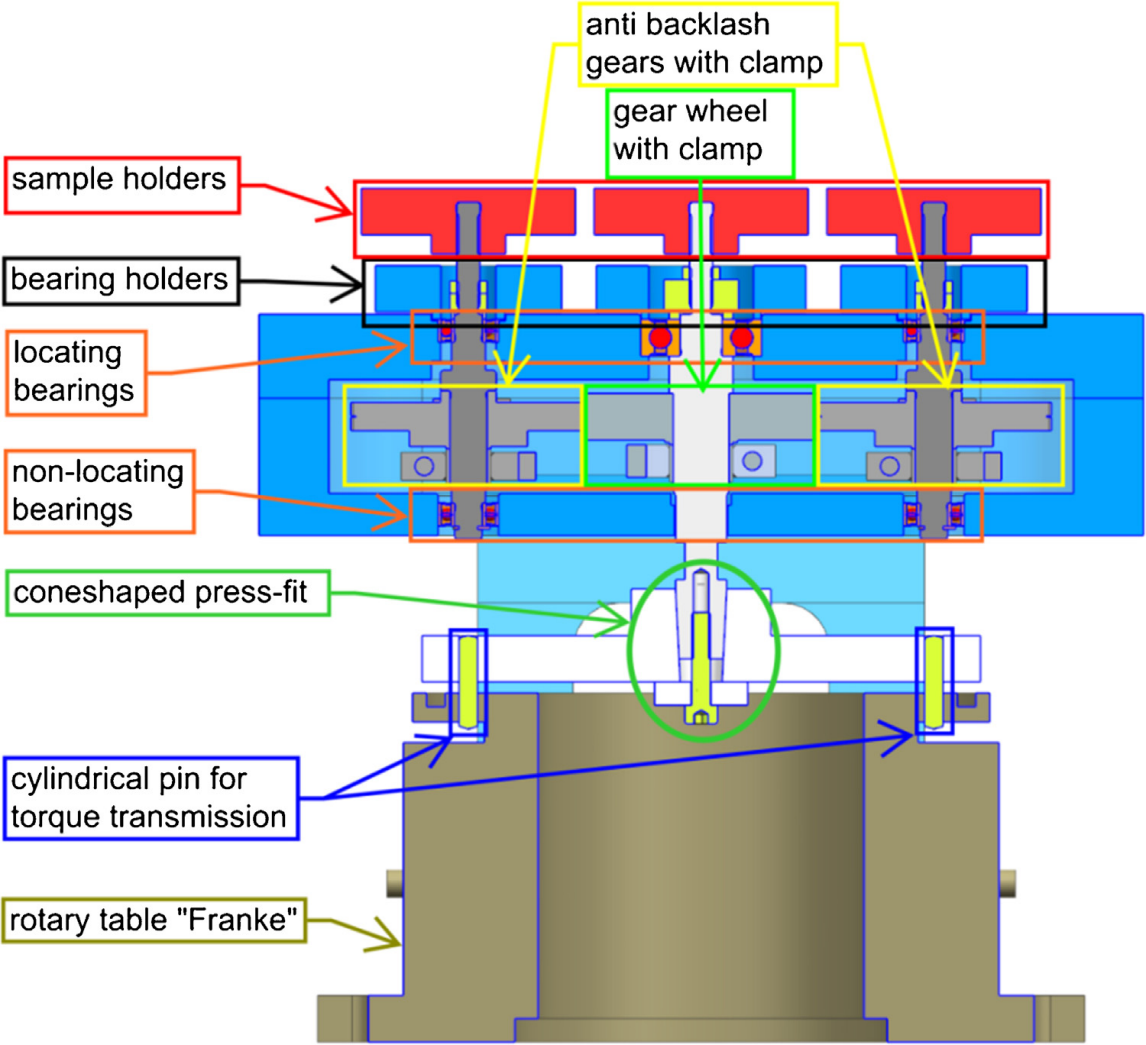
Cross-section of the rotation axis demultiplexer (POLYTOM): the device is placed on the existing rotary table (only inner parts shown). The key components of the setup are the anti-backlash gears and the stiff and precise alignment of all parts. Both concepts contribute to the precise movement of the axes and thus the sample holders.

The details of the design are shown in Fig. 2. The torque is supplied by the existing rotational stage. It is then transferred to the setup by cylindrical pin press fits that are designed to minimize the tolerances at this position. The tolerances are further reduced by the cone-shaped press fit, which transfers the torque to the input stage. The core of the demultiplexer consists of a backlash-free gear set. While the input axis is connected to the existing rotational stage and fitted with a standard gear, the output axes are fitted with clamp hub anti-backlash gears (Reliance Precision Ltd., Huddersfield, UK). Due to the gear arrangement, the output stages of the POLYTOM device rotate counter-clockwise, in the opposite direction of the input stage. The two anti-backlash gears are tensioned internally with a spring such that one of the two gears is always in contact with the gear of the central axis and thereby eliminating any potential backlash. The torque is transferred from the anti-backlash gears to the output axes through clamps. The rigidity and precision of the setup is further enhanced by mounting the side axes with a combination of locating and non-locating bearings (mounted with bearing holders) in the upper and lower housings. The precise alignment of the two housings is obtained through the output stages and the bearings holding them in place. Finally, the sample holders are firmly screwed onto the axes, thus minimizing movement of the sample. Regarding the allowable load, the output stages were designed in such a way that their load capacity is at least 2.5 kg.

**Fig. 3:**
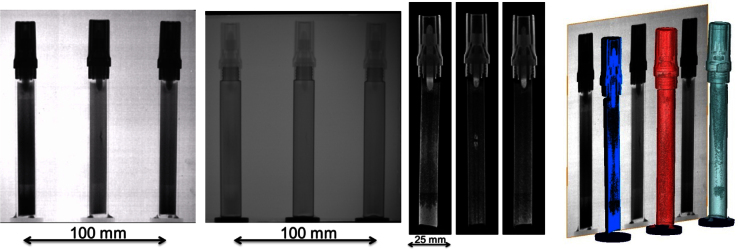
Results of the pilot experiment using the rotation axis demultiplexer (POLYTOM): Neutron radiography of three whiteboard markers (far left), corresponding X-ray radiography (left), reconstructed vertical slices from neutron tomographic datasets showing clear differences in the distribution of ink inside the whiteboard markers (right), 3D renderings of the neutron tomographic datasets showing the distribution of predominantly hydrogenous materials) in the whiteboard markers (far right).

Three whiteboard markers were used as our replicate samples and were simultaneously tomographed using POLYTOM at the NEUTRA beamline [9]. We acquired 375 projections over a 180° rotation with 5 open beam and 5 dark current images. The exposure time for each projection was 22 s, giving a total time of approximately 2 h and 20 min. The images covered a field of view of approximately 133 mm × 133 mm in size, while the pixel size was 65 μm. The acquired images were cropped and tomographic datasets of each object were reconstructed individually. An X-ray radiography image was also taken using the same detector arrangement to compare neutron and X-ray imaging modalities. Images in Fig. 3 show the comparison of neutron and X-ray radiographies of the test sample acquired with the POLYTOM device. The images confirm that the device is stable and that the reconstructed images do not exhibit any artifacts related to the use of the POLYTOM.

## Discussion

Our pilot experiment shows that the POLYTOM is capable of reducing the beam-time allocation needs for a multi-sample experiment by a factor of three. We envision that a device with even more than three parallel axes could be designed and constructed; however, such a device would likely have much more stringent requirements for fabrication and assembly. Namely, as gears become smaller, the bearings also become smaller. This means that at a certain point special bearings (e.g. those developed for the use in watch industry) would have to be used.

Given that the amount of time required for tomographic experiments increases with the enhancement in the spatial resolution, we foresee that a device similar to POLYTOM could be downscaled and also be used to create images at higher spatial resolutions [11], [12]. Current commercially-available backlash-free gears allow for a minimum spacing of about 11 mm between the parallel axes; therefore, a similar device could be downscaled nearly by a factor of 5 without major changes in design, fabrication, or assembly. However, as mentioned previously, the precision requirements for the fabrication (i.e. tolerances) and assembly of the device (e.g. drilling holes) will be correspondingly higher. On a similar topic, even the current version of POLYTOM device could be possibly used for imaging with higher spatial resolution, providing the pixel detector with larger number of pixels (e.g. 4096 × 4096 pixels) is used in conjunction with appropriate optics and with thinner neutron sensitive scintillators. Necessarily, the stability issues would have to be checked again using such a higher resolution imaging set-up.

Finally, even though the demultiplexer has been developed for the purpose of neutron tomographic imaging, it can naturally be used for other imaging types. This is especially true for imaging types that are not extensively limited by the low fluxes (e.g. X-ray tomography). Likewise, we expect that the POLYTOM device could be used to observe three different processes on three replicate samples simultaneously.

## Conclusion

We developed a rotation axis demultiplexer that permits simultaneous computed tomography of multiple samples. In the pilot experiments, we demonstrated that the device provides satisfactory results. We show that the stability of the adjacent axes of the device is sufficient to guarantee the same spatial resolution of the tomographic reconstructions for all the samples tested. As a consequence, we show that the demultiplexer is capable of reducing the beam-time allocation needs for a multiple sample imaging experiments by a factor of three.
